# Supplementary material for: Absence of Adiponutrin (PNPLA3) and Monoacylglycerol Lipase Synergistically Increases Weight Gain and Aggravates Steatohepatitis in Mice
Source: Int J Mol Sci. 2021 Feb 20;22(4):2126. doi: 10.3390/ijms22042126 (PMC7924608; doi:10.3390/ijms22042126)
Supplement: Supplementary file 1 [file ijms-22-02126-s001.pdf]

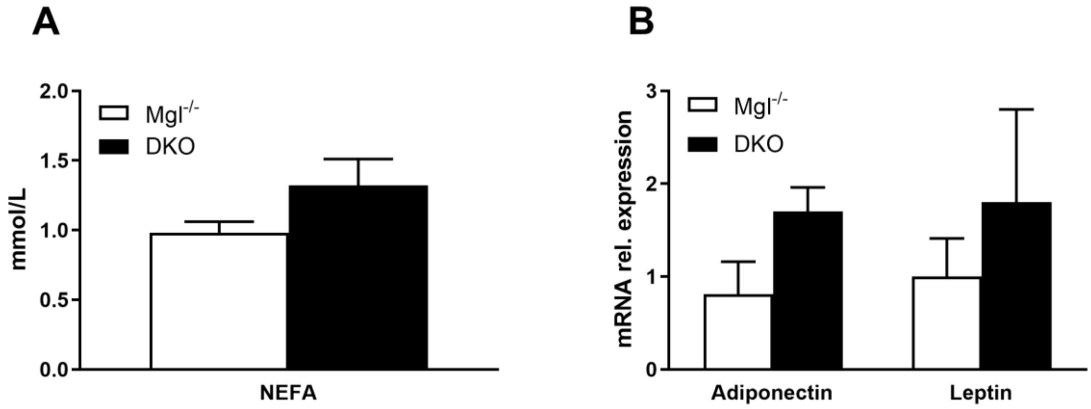

Supplemental Fig. 1. (A) NEFA were measured in sera from *Mgl*$^{-/-}$ and *DKO* mice. (B) *Adiponectin* and *leptin* gene expression were measured in AT from *Mgl*$^{-/-}$ and *DKO* mice fed HFD.
